# The Choice of Diet Affects the Oral Health of the Domestic Cat

**DOI:** 10.3390/ani5010101

**Published:** 2015-02-16

**Authors:** Fernando Mata

**Affiliations:** School of Agriculture, Food and Rural Development, Newcastle University, Newcastle upon Tyne NE1 7RU, UK; E-Mail: fernando.da-mata@newcastle.ac.uk; Tel.: +44-191-222-6870

**Keywords:** cat, diet, oral hygiene, periodontal disease, teeth

## Abstract

**Simple Summary:**

Oral health was assessed in different teeth of 41 cats of different ages and diets. It was found that oral health in cats varies with the variables considered. Incisors of young or adult cats, fed a dry diet, had better health in comparison to cheek teeth of older cats fed a wet diet. It is argued that cats’ oral health may be promoted with an early-age cheek teeth hygiene and provision of abrasive dry food.

**Abstract:**

In this cross-sectional study, the gingivitis and the calculus indices of the teeth of *N* = 41 cats were used to model oral health as a dependent variable using a Poisson regression. The independent variables used were “quadrant”, “teeth type”, “age”, and “diet”. Teeth type (*p* < 0.001) and diet (*p* < 0.001) were found to be significant, however, age was not (*p* > 0.05). Interactions were all significant: age x teeth (*p* < 0.01), age × diet (*p* < 0.01), teeth × diet (*p* < 0.001), and teeth × age × diet (*p* < 0.001). The probability of poor oral health is lower in the incisors of young or adult cats, fed a dry diet in comparison to the cheek teeth of older cats fed a wet diet. Diet has a higher contribution to poor oral health than age. It is argued that cats’ oral health may be promoted with an early age hygiene of the cheek teeth and with provision of abrasive dry food.

## 1. Introduction

Periodontal disease (PD) has been recognised as one of the most prevalent diseases in cats, affecting around 70% of the domestic cats over two years of age [1], and 85% of those aged over five years [2]. PD is a generic term of a plaque-induced inflammatory condition, affecting the periodontium [3]. The aetiology of this condition starts with the formation of dental plaque that extends into the gingival sulcus and, with the aid of the calcium salts from saliva, produces the calculus, which is the main cause for the development of gingivitis [4]. Plaque is a layer of microorganisms, mostly bacteria, adhered to the teeth, and is responsible for the initiation of PD [5]. PD initially begins with gingivitis, which can then develop into periodontitis if left untreated. While gingivitis is reversible by treatment, PD of brachydont teeth is irreversible and can only be managed to avoid further progression, once irreversible destruction of connective tissues and loss of adjacent bone has taken place [4,6]. Systemic disease has been increasingly recognised in cats affected by PD [1,4]. Research suggests that PD has an association with the development of cardiorespiratory, hepatic, and renal disorders [4], and also diabetes mellitus in humans [7].

Calculus formation and the development of gingivitis are key aspects in the development of PD; as calculus and gingivitis increase, oral health deteriorates. A relationship between the degree of calculus and gingivitis development signals, therefore, a deterioration of the teeth health status. The calculus index (CI) proposed by Ramfjord [8], and the gingival index (GI) proposed by Loe and Silness [9] are still used today to assess the degree of development of these two conditions and can, therefore, be used to assess the oral health of cats. The GI scoring criteria are: 0 (normal), 1 (mild inflammation, slight colour change, slight oedema, no bleeding on palpation), 2 (moderate inflammation, redness, oedema, bleeding on probing), and 3 (severe inflammation, marked redness and oedema, tendency to spontaneous bleeding). The CI scoring criteria are: 0 (no calculus present), 1 (supra gingival calculus covering one third of the exposed tooth surface), 2 (supra gingival calculus covering more than one third to two thirds of the exposed tooth surface or presence of flecks of sub gingival calculus, or both), 3 (sub gingival calculus covering more than two thirds of the exposed tooth surface or a continuous heavy band of sub-gingival calculus around the crevices of teeth or both).

The prevalence and severity of PD varies with several factors, such as: gender, age, breed, diet, chewing behaviour, and systemic health [6]. Several studies have related the type of diet and age with the development of PD (e.g., [4,6,10]) but the relationship between these factors and the type of teeth has not yet been considered. Watson [4] performed a revision of the literature on the relationship between PD and diet in dogs and cats, and argues that the main advances in diet formulation for these animals have improved their health, especially in relation to nutritional deficiencies; on the other hand he points out the importance of the physical properties of the diet (texture, abrasiveness and chewiness) as additional methods to control plaque and prevent PD. Clarke and Cameron [6] compared the development of calculus and PD in domestic and feral cats in Australia, and found that calculus develops easily in domestic cats but no differences were found for PD. Once domestic cats were being fed with a canned and dry diet, they concluded that the live prey-based diet may prevent the development of calculus. Gawor *et al.* [10], while studying the influence of diet on oral health of dogs and cats, concluded that dental calculus and plaque were less frequent in cats fed dry, rather than wet, food. These authors also observed a positive correlation between age and calculus formation.

Previous studies considered the factors of age and type of diet individually, without looking into the type of teeth and into the interactive effects. The aim of this study is to verify the interactive effects of age, type of diet, and type of teeth, and to develop a stochastic model to allow the prediction of the impact on the oral health and welfare of cats.

## 2. Experimental Section

In this cross-sectional study, data were collected within Pet Doctors™ Veterinary Hospital on the Isle of Wight in England, taken from *N* = 41 Domestic Short Hair cats, during January 2013. The study did not require approval from an Ethics Committee, as it was based in data collected from normal clinical practice. Pet Doctors™ Veterinary Hospital is part of the Corporate Veterinary Surgeons (CVS UK) Limited and complies with the ethical rules and regulations on the treatment of animals set by the British legislation and professional bodies. All the cats used in the study are healthy animals, presented for surgery for other than the reasons of disease or illness: routine worming or check-up, spaying, or a simple post-traumatic procedure. All cats in this study had oral home care.

The gingivitis index (GI) and the calculus index (CI) were assessed for each of the cat’s teeth, always by the same veterinary surgeon to avoid assessors’ bias. The examination was done with the cat awake, without use of any medication or anaesthesia. None of the cats was subjected to a frequent type of dental hygiene. The cat owners were asked to fill in a questionnaire concerning their pet, having been explained the purpose of the questionnaire. Questions asked: “age” (young—up to 3 years, adult—from 3 to 8 years, and old—more than 8 years) and predominant “diet” (commercial dry, commercial wet, mixed commercial dry and wet, homemade).

**Figure 1:**
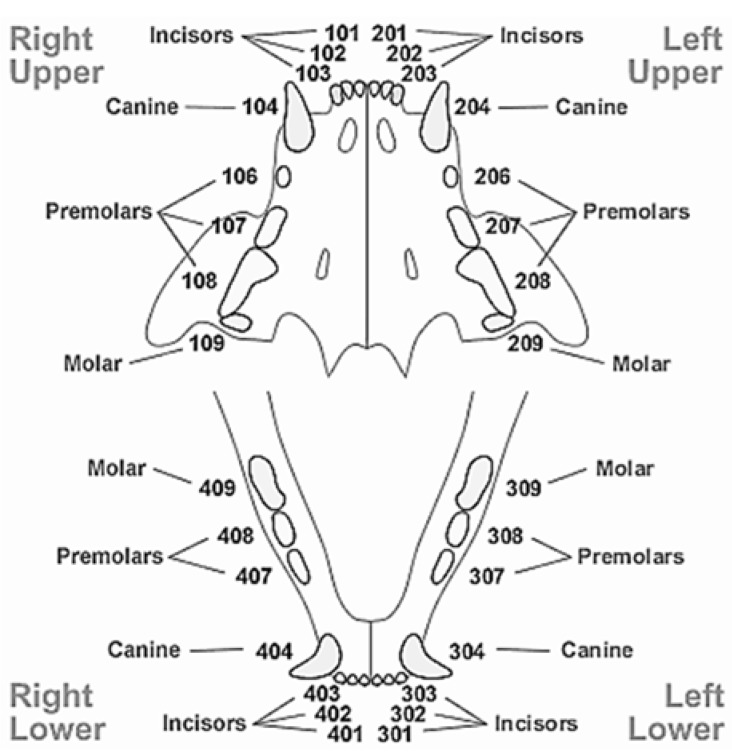
The TRIDAN modified system for cat teeth classification. Adapted from Crossley [12].

Data were organised by quadrant (“side”—left or right, and “position”—maxilla or mandible) and “type of teeth” (incisors, canines, premolars, and molars). Teeth nomenclature used the TRIDAN modified system [11], according to Figure 1. Averages for CI and GI were calculated between: 101, 102, and 103 for upper right incisors; 201, 201, and 203 for upper left incisors; 301, 302, and 303 for lower left incisors; 401, 402, and 403 for lower right incisors; 106, 107, and 108 for upper right premolars; 206, 207, and 208 for upper left premolars; 307 and 308 for lower left premolars; and 407 and 408 for lower right premolars. For canines and molars, the values entered were those assessed as only one of these tooth types exist per quadrant. Finally, GI and CI were added together to create the variable teeth health status (THS). Values were rounded to the nearest unit and ranged from 0 to 6, as both CI and GI ranged from 0 to 3.

A generalised estimating equation approach was used to account for the within-subject effects of the variables side, position and type of teeth. A multinomial cumulative logit link function was fitted using THS as the dependent variable. The variables of age, type of teeth (incisive, canine, premolar, molar), and diet (dry, wet, mixed, homemade) were entered in the analysis as factors; first and second level interactions between factors were also entered. A type III sum of squares was chosen once the analysis had an unbalanced design. The variables were entered in the model following a forward stepwise procedure and were tested using the Wald Chi-square statistic, with the significance level set to *p* < 0.05. The analysis was done via generalised linear models routine, using the software IBM^®^ SPSS^®^ Statistics for Windows^®^, version 21.0. (IBM Corp., Armonk, NY, USA, 2012).

## 3. Results and Discussion

### 3.1. Results

From the factors analysed, teeth (*p* < 0.001) and diet (*p* < 0.001) were shown to be significant. Age was not significant (*p* > 0.05) as a stand-alone factor, however it was found significant within first and second level interactions, together with the 1st and 2nd order interactions between all the other factors: age × teeth (*p* < 0.01), age × diet (*p* < 0.01), teeth × diet (*p* < 0.001), and teeth × age × diet (*p* < 0.001).

Table 1 shows the parameters of the six equations for the calculation of probabilities of observation of a particular THS score, given a particular combination of factors. These equations have the generic formula:

(1) $$P\left(THS_{n}\right)=\frac{\text{exp}({\text{β}}_{0n}+{\text{β}}_{1}+{\text{β}}_{2}+{\text{β}}_{1,2}+{\text{β}}_{1,3}+{\text{β}}_{2,3}+{\text{β}}_{1,2,3})}{1+\text{exp}({\text{β}}_{0n}+{\text{β}}_{1}+{\text{β}}_{2}+{\text{β}}_{1,2}+{\text{β}}_{1,3}+{\text{β}}_{2,3}+{\text{β}}_{1,2,3})}$$

where *P*(*THS_n_*) is the probability of a particular THS (*n* scoring 1 to 6), β*_0n_* is the threshold parameter for each one of the six equations (*n* scoring 1 to 6), β*_1_* is the parameter for type of teeth, β*_2_* is the parameter for diet, β*_1,2_* is the parameter for teeth × diet, β*_1,3_* is the parameter for teeth × age, β*_2,3_* is the parameter for diet × age, and β*_1,2,3_* is the parameter for teeth × diet × age.

**Table 1:** Parameters estimation for the equations used to calculate the probability of each of the combinations of teeth type, diet, and age, to score each of the THS values.

Variable		Parameter Estimation (β)	SE
Threshold	THS_6_	−3.391	1.408
	THS_5_	−2.492	1.334
	THS_4_	−1.166	1.287
	THS_3_	0.154	1.263
	THS_2_	1.680	1.233
	THS_1_	2.432	1.280
Teeth	Incisors	1.948	2.184
	Canines	0.767	1.777
	Premolars	−2.993	1.927
	Molars	0	
Diet	Dry	−0.051	1.477
	Wet	−1.826	1.451
	Dry + Wet	1.686	0.618
	Homemade	0	
Incisors × Young		−0.121	1.7749
Incisors × Adult		0.699	1.1050
Canines × Young		0.150	1.3088
Canines × Adult		0.091	0.9347
Premolars × Young		2.402	1.3880
Premolars × Adult		2.263	0.9023
Molars × Young		−0.477	1.5227
Molars × Adult		−2.035	1.2142
Young × Dry		2.138	2.080
Young × Wet		1.298	2.176
Young × Dry + Wet		−3.038	1.063
Adult × Dry		3.010	1.511
Adult × Wet		2.107	1.463
Incisors × Dry		1.220	2.345
Incisors × Wet		2.914	2.402
Incisors × Dry + Wet		−2.241	1.308
Canines × Dry		−0.175	1.833
Canines × Wet		0.434	2.071
Canines × Dry + Wet		−1.898	0.938
Premolars × Dry		2.553	2.061
Premolars × Wet		2.125	2.045
Premolars × Dry + Wet		−0.864	0.625
Incisors × Young × Dry		−1.440	2.945
Incisors × Young × Wet		−1.593	2.963
Incisors × Young × Dry + Wet		5.461	2.191
Incisors × Adult × Dry		−2.942	2.489
Incisors × Adult × Wet		−5.459	2.543
Canines × Young × Dry		0.729	2.089
Canines × Young × Wet		−0.255	2.481
Canines × Young × Dry + Wet		0.505	1.227
Canines × Adult × Dry		−0.755	1.916
Canines × Adult × Wet		−2.343	2.136
Premolars × Young × Dry		−1.768	2.581
Premolars × Young × Wet		−1.226	2.628
Premolars × Young × Dry + Wet		0.978	1.387
Premolars ×Adult × Dry		−3.023	2.075
Premolars × Adult × Wet		−3.016	2.060

Table 2 gives the different odds ratio readings as probabilities of scoring each of the THS for the different combinations of type of teeth, diet, and age. These are the probability values calculated after application of the generic formula previously introduced.

The odds ratio increases with the value of the parameter, therefore, as can be observed, and as expected, the odds ratios for a lower score are higher than those of a higher score (threshold for THS_1_ = 2.432 and for THS_6_ = −3.391, with the others ordered in between). This principle could be applied to the variables in the model, provided that no interaction was observed; once this was not the case, the odds ratio for the different variables needs to be contextualised within the interaction.

**Table 2:** Probabilities of THS score in dependency on the different combinations between the levels of the factors analysed (teeth, age, diet). Probabilities are in ascending order to aid reading.

Variables			Probabilities for the Different THS Scores					
Teeth	Age	Diet	6	5	4	3	2	1
incisors	adult	dry	0.001	0.002	0.006	0.023	0.099	0.190
incisors	young	dry	0.001	0.002	0.008	0.028	0.118	0.221
incisors	young	dry + wet	0.001	0.002	0.008	0.028	0.118	0.221
canines	young	dry	0.001	0.002	0.009	0.032	0.133	0.245
incisors	old	dry	0.001	0.004	0.014	0.049	0.192	0.335
incisors	old	wet	0.002	0.004	0.015	0.053	0.205	0.353
canines	adult	dry	0.002	0.005	0.017	0.061	0.230	0.388
incisors	adult	homemade	0.002	0.006	0.022	0.076	0.275	0.446
incisors	young	wet	0.002	0.006	0.022	0.078	0.281	0.453
premolars	young	dry	0.003	0.008	0.031	0.106	0.354	0.538
incisors	adult	dry + wet	0.004	0.010	0.037	0.126	0.398	0.584
incisors	young	homemade	0.005	0.013	0.048	0.158	0.463	0.647
premolars	adult	dry	0.006	0.014	0.051	0.167	0.480	0.662
molars	old	dry + wet	0.006	0.015	0.055	0.178	0.498	0.678
molars	young	dry	0.007	0.016	0.059	0.189	0.517	0.695
incisors	old	dry + wet	0.008	0.020	0.072	0.225	0.571	0.739
molars	adult	dry	0.013	0.032	0.110	0.316	0.680	0.819
canines	young	homemade	0.013	0.032	0.111	0.318	0.682	0.820
canines	adult	homemade	0.014	0.034	0.117	0.331	0.695	0.828
canines	adult	dry + wet	0.017	0.042	0.140	0.379	0.738	0.856
canines	young	wet	0.019	0.045	0.150	0.398	0.753	0.866
canines	old	dry + wet	0.019	0.045	0.152	0.401	0.755	0.867
canines	old	dry	0.019	0.046	0.154	0.404	0.757	0.869
incisors	adult	wet	0.022	0.053	0.175	0.443	0.785	0.886
premolars	adult	dry + wet	0.030	0.070	0.221	0.515	0.830	0.912
molars	old	dry	0.034	0.080	0.247	0.551	0.850	0.923
premolars	young	wet	0.040	0.094	0.280	0.592	0.870	0.934
molars	adult	dry + wet	0.046	0.105	0.306	0.623	0.884	0.942
molars	young	homemade	0.051	0.118	0.334	0.653	0.896	0.948
premolars	old	dry	0.052	0.119	0.337	0.656	0.898	0.949
premolars	young	homemade	0.057	0.130	0.360	0.678	0.906	0.954
canines	old	wet	0.059	0.134	0.368	0.685	0.909	0.955
premolars	adult	homemade	0.065	0.147	0.393	0.708	0.918	0.959
canines	adult	wet	0.068	0.152	0.402	0.716	0.921	0.961
molars	young	wet	0.084	0.184	0.460	0.761	0.936	0.969
premolars	adult	wet	0.114	0.24	0.543	0.817	0.953	0.978
molars	adult	wet	0.163	0.323	0.643	0.871	0.969	0.985
molars	old	wet	0.173	0.339	0.659	0.879	0.971	0.986
canines	young	dry + wet	0.173	0.340	0.660	0.879	0.971	0.986
premolars	young	dry + wet	0.173	0.340	0.660	0.879	0.971	0.986
molars	young	dry + wet	0.173	0.340	0.660	0.879	0.971	0.986
molars	adult	homemade	0.205	0.388	0.705	0.899	0.976	0.989
premolars	old	dry + wet	0.228	0.420	0.732	0.911	0.979	0.990
premolars	old	wet	0.332	0.550	0.822	0.945	0.988	0.994

THS: teeth health status; Note: Probabilities including the combinations of “old age” and “homemade diet” were not computed as this combination was inexistent in the sample.

### 3.2. Discussion

In a very generic way, it can be observed that incisors of young or adult cats with a dry diet have lower probabilities of scoring a high TSH value, in comparison to the premolars and molars (cheek teeth) of older cats having a diet with a wet component. Incisors score even lower in older cats, independently of type of food. Cheek teeth score higher, even in young cats, but predominantly where wet commercial food or homemade food (which is also wet/soft) was fed. Age is shown to be the least predominant factor of the three considered. Diet and type of teeth were found to be the predominant and determinant factors responsible for the variability in scores, and, therefore, cat oral health status.

Cheek teeth are larger and hide in the buccal cavity and are difficult to be cleaned by the tongue, which explains the accumulation of food debris, which builds up the bacterial pool and encouraging the development of both plaque and gingivitis at higher frequencies. Cheek teeth also play a predominant role in mastication, and, as the incidence of diastemata in these teeth is more common than in the incisors, the creation of food pockets may prevail. One last aspect differentiates cheek teeth and incisors in cats: the role played by incisors in corporal hygiene, which contributes to a higher degree of abrasion and, therefore, the prevention of plaque formation.

Several authors have previously identified wet or soft food as being responsible for a lower health status of teeth [4,10,13,14] when compared to dry food. The explanation for this fact stays with the abrasive properties of dry food, capable of removing the teeth plaque. Clarke and Cameron [6] have studied the impact of a commercial diet (mixed, wet, and dry) in domestic cats, by comparing them with their feral cat free hunter cousins, but found no significant differences between them in calculus formation and degree of PD. These authors concluded that commercial food cannot be solely responsible for the development of PD in cats. However, Gorrel *et al.* [15] demonstrated the beneficial effect of the addition of a “dental hygiene chew” feed in terms of prevention of plaque and calculus accumulation on tooth surfaces, even when cats were fed dry food.

Dry food can eventually be responsible for an increased production of saliva. It is well known that saliva contains immunoglobulins produced in reaction to the antigens found in the mouth [16], and, therefore, dry food eventually will also be responsible for a better use of the immune system in the prevention of oral health issues.

Good oral hygiene has proven to prevent the development of PD in cats, and several authors refer to that in review articles (e.g., [3]) and research articles after trials (e.g., [1]). It has been identified that it is difficult to habituate a cat to dental hygiene methods and it has been suggested that an early habituation, from the kitten stage, is fundamental. The results of the present study show that, even at an early age, cats are susceptible to poor health status in their cheek teeth, especially if eating wet food. It is suggested that good hygiene of the feeding bowl should also be considered to avoid bacterial build up, especially where cats are fed wet food, as bacteria is the main trigger of plaque development, leading to PD.

As a limitation in this study, we must refer that several other factors may eventually play an important role in cats’ oral hygiene. For example, Kornya *et al.* [17] have recently established a relationship between the deterioration of cats’ oral health and the positive testing for retroviruses (feline leukaemia virus and feline immunodeficiency virus), and cats in this study were not tested for retroviruses. In addition, homemade diet nutritional details may vary substantially (nutritional deficiencies, feeding of bones, *etc.*), which may impact on results.

Water intake is another factor with a possible impact on cats’ oral health, but the question is not addressed by this study. Cats are animals with a desert origin and the moisture content of food is an important part of water intake. While the moisture content of wet food is over 75%, dry food contains around 6% [18]. Cats fed wet food voluntarily drink small quantities of water while cats fed dry food have a higher voluntary intake [19], which, however, does not compensate for the reduced moisture content [20].

## 4. Conclusions

Cheek teeth (molars and premolars) are more susceptible to poor oral health than other teeth, independent of the age of the cat. It is important to prevent oral health deterioration from an early age with special attention paid to the cheek teeth. The diet of a cat needs to be considered holistically, paying particular attention to its nutritional value, but the texture of the food is shown to play an important role in oral health, with wet canned food providing the least benefit to oral health.
